# Diversity of the microbial community and antioxidant activity during fermentation of red raspberry Enzymes

**DOI:** 10.1002/fsn3.1961

**Published:** 2020-12-22

**Authors:** Di Yao, Lei Xu, Changyuan Wang

**Affiliations:** ^1^ Department of Food Science and Engineering College of Food Heilongjiang Bayi Agricultural University Daqing China; ^2^ Department of National Coarse Cereals Engineering Research Center Heilongjiang Bayi Agricultural University Daqing China

**Keywords:** antioxidant activity, microbial diversity, red raspberry Enzymes

## Abstract

The diversity and succession of microbial community and antioxidant activity present during the preparation of red raspberry Enzymes with and without starter cultures were investigated by high‐throughput sequencing of 16S rRNA and ITS1 genes and correlation analysis of the microbial diversity and antioxidant activity. The results showed that the sample inoculated with mixed fermentation had higher antioxidant activity than the sample without inoculated fermentation. The antioxidant capacity of red raspberry Enzymes increased significantly as the fermentation time increased. Firmicutes and Ascomycota were the predominant phyla of bacteria and fungi in all samples. At the genus level, *Rhodococcus* and *Lactobacillus* were the predominating bacteria throughout the fermentation process. The genus *Kodamaea* dominated the fungal community of early‐fermentation samples with microbial inoculated fermentation. *Candida* spp. grew rapidly in the late stage of fermentation in the samples with spontaneous fermentation. Unweighted pair‐group and PCA analysis revealed that the microbiota structures differed between the two groups. RDA and CCA showed that *Rhodococcus* and *Kodamaea* had positive effects on the DPPH scavenging ability and other antioxidant indicators, and the total phenol content had a significant and positive correlation coefficient with *Gluconobacter*. The results indicated that the fermentation by microorganisms significantly improves the oxidation resistance and helps to improve the quality of the red raspberry Enzymes.

## Graphical abstract

Red raspberry Enzymes inoculated with mixed fermentation had higher antioxidant activity than the sample without inoculated fermentation. The microbiota structures differed between the two samples. The results indicated that the fermentation by microorganisms significantly improves the oxidation resistance and helps to improve the quality of the Enzymes.

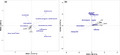

## 1 INTRODUCTION

Enzymes are a kind of plant‐derived functional food that is common all around the world, especially popular in Japan, the United States, and Taiwan. One or more fresh fruits, vegetables, mushrooms, and other pure natural plants are used as raw materials for spontaneous fermentation or include starter cultures for fermentation. Enzymes are rich in nutrients, such as phenol and organic acids produced by microbial fermentation, which possess antioxidant effects and can improve immunity, gastrointestinal health, etc. (You & Binin, 2016).

In general, yeast, lactic acid bacteria, and acetic acid bacteria are used as starters in fermented food. Fruits and vegetables fermented by lactic acid bacteria and yeast can not only retain the original nutrients but also bring many benefits, such as improving human digestibility and providing antioxidant effects (Komatsuzaki et al., 2005). At present, many scholars are committed to antioxidant activity research on fruit Enzymes, for example, Zhiqiao et al. studied DPPH free radical, hydroxyl free radical, and total antioxidant capacity in pineapple Enzymes, and the results showed that pineapple Enzymes has good antioxidant properties (Qiao et al., 2020). Four kinds of black fruit medlar as raw materials were used to analyze the active components and antioxidant activities before and after fermentation; the results showed that the antioxidant activity of fermented blackberry wolfberry is higher than that of unfermented blackberry wolfberry (Chao et al., 2019). Therefore, the action of microorganisms in Enzymes can enhance the antioxidant activity of this product. However, researchers have mainly focused on the fermentation of various advantageous microorganisms to produce Enzymes products, and there is a lack of studies on the mechanism of changes in the antioxidant activity by microbial fermentation of Enzymes; likewise, there are few studies on the correlation between the microbial diversity and antioxidant activity in Enzymes.

The red raspberry is a berry of the shrub *Rubus idaeus* L, whose output is 400,000 tons per year worldwide. Many reports have shown that red raspberry has a positive impact on human health in view of its functional ingredients including flavonoids and polyphenols (Rios et al., 2014); moreover, the active ingredients have good antioxidant properties. However, little attention has been paid to the processed products of red raspberries. In recent years, there have been a few studies on red raspberry Enzymes, but the correlation between the antioxidant activity and microbial diversity of this product has not been reported. So far, high‐throughput sequencing (HTS) methods have become indispensable tools for the detection of microbial community structure and composition (Camilla et al., 2017). HTS has high efficiency and sensitivity; moreover, it can detect dominant and low‐abundance microorganisms in a sample and can accurately reflect the diversity of microorganisms in the sample at different fermented periods.

The accumulation of antioxidant substances in fermented foods is a result of a complex process that is affected by multiple factors and their interactions; undoubtedly, the action of microorganisms in Enzymes plays a significant role in the antioxidant activity. Nevertheless, the relationship between the accumulation of antioxidant substances and the core microbial population in Enzymes at different fermentation periods is still not well known. In this study, we produced two groups of red raspberry Enzymes via spontaneous fermentation and microbial inoculated fermentation. A comprehensive analysis of the antioxidant activity in red raspberry Enzymes at different fermentation stages was performed, including the DPPH and hydroxyl radical scavenging ability, SOD activity, total phenol content, and total reducing power. Moreover, the HTS method was chosen to analyze the microbial diversity and community. Finally, the correlation between the two was analyzed, which could not only evaluate the structure of microbial communities and the antioxidant activity at different fermentation stages of Enzymes (Gülay et al., 2016) but also reflect the mechanism and response characteristics of Enzymes in specific ecosystems to cope with current and future environmental changes (Salis et al., 2017). Therefore, the correlation analysis of the microbial diversity and antioxidant activity can provide a reliable theoretical basis for the development and research of Enzymes and promote the application of mixed strain fermentation in functional Enzymes products.

## 2 MATERIALS AND METHODS

### 2.1 Starter cultures

Strains of *Lactobacillus plantarum* (*L. plantarum*) DQ‐13 and *Kodamaea ohmeri* (*K. ohmeri*) WP‐1 were isolated from pitaya Enzymes in our previous study. *Lactobacillus plantarum* DQ‐13 was cultivated twice in DeMan‐Rogosa‐Sharpe (MRS) broth at 37°C for 24 hr; *K. ohmeri* WP‐1 was cultivated twice in yeast extract peptone dextrose medium (YPD) broth at 30°C for 2 days. Subsequently, all the cells were centrifuged at 11,000 *g* and washed using sterile saline water. Finally, the cell concentrations of *L. plantarum* DQ‐13 and *K. ohmeri* WP‐1 were determined by the plate counting method and adjusted to 5 × 10^8^ cfu/ml and 1 × 10^7^ cfu/ml using sterile water.

### 2.2 Enzymes manufacturing and sampling

A total of two copies of red raspberry (average weight 2 ± 0.1 kg) samples were purchased from Hailin, China. The samples were kept in sterile plastic bags at 4°C, transported to the laboratory within 12 hr and then cleaned using sterile water before fermentation. Enzymes was prepared according to the reported method (Jin et al., 2020) with small modifications. First, 2 kg of crystal sugar was added to red raspberries and fully dissolved. Subsequently, the mixtures were randomized into two groups. One group was fermented spontaneously and called spontaneous fermentation (S), and another group was inoculated with a mixed starter culture (*L. plantarum* DQ‐13 and *K. ohmeri* WP‐1 = 1:1) at 1% inoculation and called microbial inoculated fermentation (M). All the products were fermented at ambient temperature (25°C) for 90 days in sealed containers with periodical stirring and tedding. Samples were taken from two groups for antioxidant analysis and high‐throughput sequencing analysis at 0 (fresh), 7, 15, 30, 60, and 90 days after fermentation.

### 2.3 Analysis of antioxidant activity

The Folin–Ciocalteu method was performed to measure the total phenol content of Enzymes by consulting the method of Zhuo et al. (2016) with some modifications. For the determination of the DPPH radical scavenging rate and SOD activity, we referred to the method of Qing et al. (2013). The hydroxyl radical scavenging rate was determined with a hydroxyl radical kit (Nanjing Jiancheng Biotechnology Research Institute, Nanjing, China) according to the manufacturer's protocol. The reducing force was determined using a Vc standard curve (Mei et al., 2015).

### 2.4 High‐throughput sequencing analysis

Total genomic DNA was extracted from 33 samples (Fresh, S7, S15, S30, S60, S90, M7, M15, M30, M60, and M90, three parallels each) with an Ezup Column Fungi Genomic DNA Purification Kit (Sangon Biotech) according to the manufacturer's protocol. The quality and quantity of the extracted DNA were assessed by the ratios of 260 nm/280 nm and 260 nm/230 nm with a Nanodrop spectrophotometer (A360, AOE). The V3‐V4 region of the bacterial 16S rRNA gene was amplified with the universal primers 338F (5′‐ACTCCTACGGGAGGCAGCA‐3′) and 806R (5′‐GGACTACHVGGGTWTCTAAT‐3′). The ITS1 region of the fungi was amplified with the primers ITS1F (5′‐CTTGGTCATTTAGAGGAAGTAA‐3′) and ITS1R (5′‐GCTGCGTTCTTCATCGATGC‐3′). The bacterial PCR amplification was performed in a total volume of 50 μl, which contained 25 μl of 2× San Taq Fast PCR Master Mix, 1 μg of sample DNA, 1 μl each of 10 μM 338F and 806R, and ddH_2_O to 50 μl as the remainder. The thermal cycling conditions were as follows: an initial denaturation at 98°C for 2 min, followed by 25 cycles at 98°C for 30 s, 50°C for 30 s, and 72°C for 40 s, with a final extension at 72°C for 5 min. The fungus PCR system (50 μl) contained 25 μl of 2× Taq PCR Master mix, 1 μg of DNA template, 1 μl each of 10 μM ITS1F and ITS1R, and ddH_2_O to 50 μl. The thermal cycling conditions were as follows: an initial denaturation at 95°C for 5 min, followed by 30 cycles at 95°C for 30 s, 50°C for 30 s, and 72°C for 40 s, with a final extension at 72°C for 7 min. The PCR products were purified using the SanPerp Column PCR Product Purification Kit (Sangon Biotech.). The 16S rRNA and ITS1 gene amplicons were sequenced using Illumina HiSeq deep sequencing v2 (Biomarker Bioinformatics Technology Co.).

### 2.5 Bioinformatics analysis

The double‐end sequence data obtained by HiSeq sequencing were spliced or merged into a sequence tag, and quality control filtering was performed on the quality of reads and the effect of merge. Then, the stitching sequence obtained is the original tag data (raw tags). The read sequences were aligned with species annotation databases through the UCHIME Algorithm (http://drive5.com/usearch/manual/uchime_algo.html), and finally, the chimera sequence was identified and removed to obtain the final valid data (effective tags). Usearch (Edgar, 2013) software was used to cluster tags at a similarity level of 97% to obtain OTUs. Alpha diversity (Chao1 richness and Ace richness estimators, Simpson and Shannon diversity indices) was evaluated using the Mothur v.1.11.0 program, and beta diversity (principal component analysis, UPGMA, heat map) was performed using QIIME software. Ultimately, redundancy analysis (RDA) and canonical correspondence analysis (CCA) were performed using Canoco software to reflect the relationship between the core microorganisms and antioxidant activity (Cruz et al., 2019).

### 2.6 Statistical analysis

The antioxidant index tests and high‐throughput sequencing of all samples were repeated three times. ANOVAs were performed using SPSS. Histograms were made with Origin 2018. The results are expressed as the mean ± *SD*, and significant differences in the two groups were confirmed at *p* < .05.

## 3 RESULTS AND DISCUSSION

### 3.1 The antioxidant activity of red raspberry Enzymes

**FIGURE 1:**
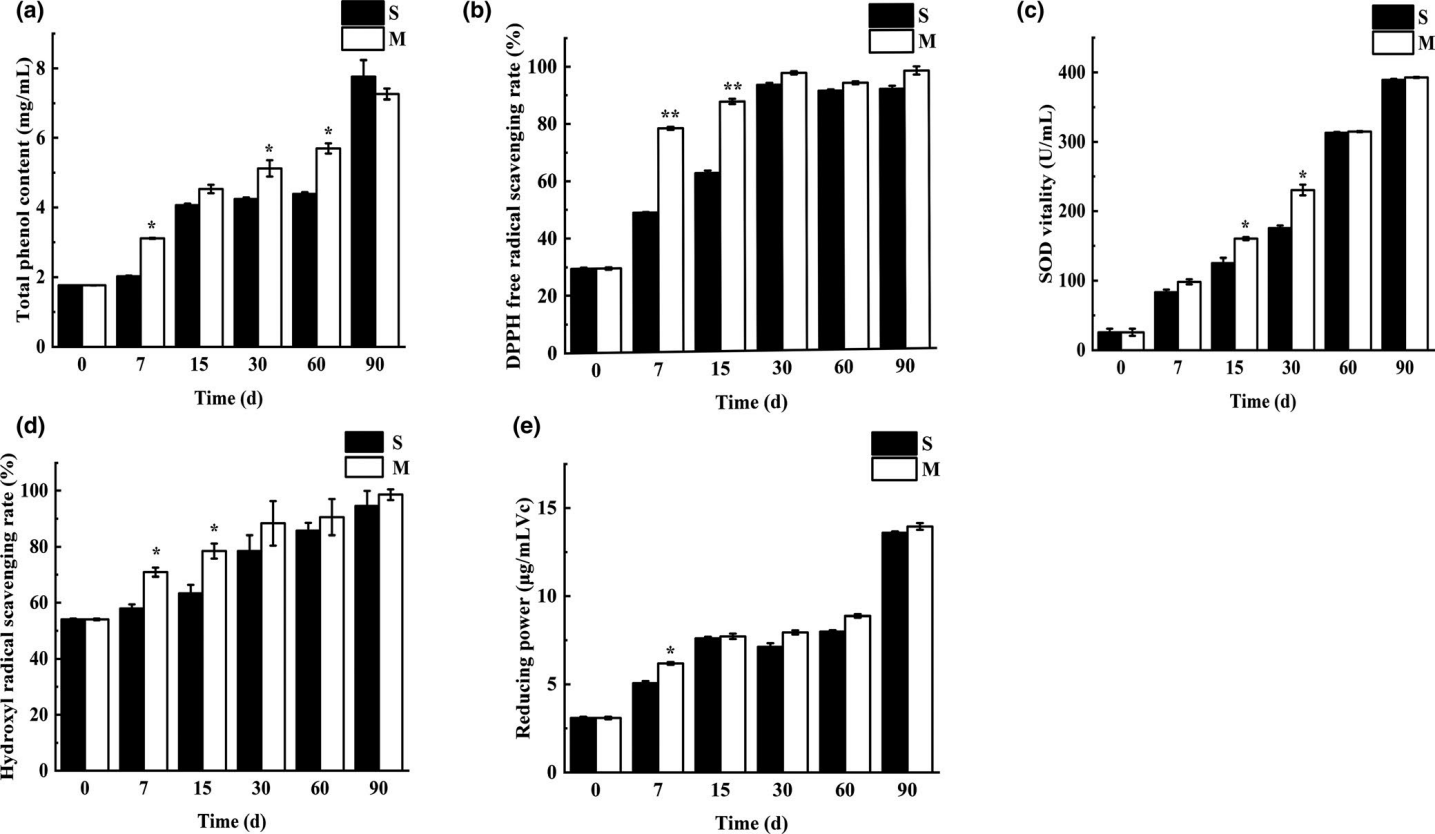
Antioxidant analysis during fermentation of red raspberry Enzymes. (a) Total phenol content, (b) DPPH radical scavenging, (c) SOD vitality, (d) Hydroxyl radical scavenging rate, (e) Reducing power. S refers to samples without starter cultures; M refers to samples with starter cultures. (7–90) indicate different fermentation stages (in days); 0 day for fresh samples. ** indicates a extremely significant difference (*p* < .01); * indicates a significant difference (*p* < .05)

In general, there is a direct correlation between total phenol content and antioxidant activity because polar or phenolic compounds can promote antioxidant activity (Orhan & Üstün, 2010). The total phenol content of the red raspberry Enzymes gradually increased during the entire fermentation process, and the content in the M group was higher than that in the S group, except for the 90 days sample (Figure 1a). Red raspberry contains a certain amount of phenolic substances, and the total phenol content was significantly increased after fermentation. Compared with those of the S group, the components of the M group were quickly decomposed under the action of microorganisms owing to the inoculated starter culture. Obviously, the role of microorganisms in increasing the total phenol content was important.

The scavenging activity of DPPH is based on one‐electron reduction, which represents the free radical reducing activity of antioxidants. As shown in Figure 1b, the DPPH scavenging ability of red raspberry Enzymes gradually increased within 30 days of fermentation, and the ability of the M group was significantly higher than that of the S group in the 7 and 15 days samples. After 30 days of fermentation, the DPPH scavenging capacity was stable at approximately 90%. In addition, the SOD activity (Figure 1c) and hydroxyl radical scavenging capacity (Figure 1d) of red raspberry Enzymes gradually increased during the entire fermentation process and were higher in the M group than in the S group, except for the hydroxyl radical scavenging ability at 30 days. Furthermore, the reducing power of red raspberry Enzymes slightly increased within 60 days of fermentation, and that at 90 days significantly increased and was the highest, but there was no significant difference between the M and S groups, except for the sample at 7 days (Figure 1e).

Combining the results and analysis, although fresh red raspberries already have a certain antioxidant capacity due to accumulated antioxidant compounds, the antioxidative activity was obviously improved after fermentation, mainly because the microorganisms can continue to accumulate antioxidant active compounds through metabolism and decomposition of macromolecular substances in the red raspberries during the fermentation process. In most cases, the antioxidative activity of the M group is significantly higher than that of the S group, illustrating that the inoculation of mixed strains has a more obvious advantage in improving the oxidation resistance of the product.

### 3.2 Sequencing and OTU cluster analysis

According to the results of sequencing and quality control processing, including paired‐read assembly and raw read filtering, a total of 2,543,082 high‐quality bacterial tags and 2,269,821 high‐quality fungal tags were obtained. Each sample was covered with an average of 77,063 bacterial tags and 68,782 fungal tags. Furthermore, based on a 97% sequence similarity level, the total numbers of operational taxonomic units (OTUs) for bacteria and fungi were 21,448 and 24,016, respectively. The number of OTUs in samples fermented spontaneously for 90 days (S90) was the highest, with 2,084 and 2,365 for bacteria and fungi, respectively (Table 1).

**TABLE 1:** Alpha diversity index of red Raspberry Enzymes

Sample	Bacterial						Fungus					
	OTU	ACE	Chao	Simpson	Shannon	Coverage	OTU	ACE	Chao	Simpson	Shannon	Coverage
F	1,363	3,241.58	2,292.21	0.4333	2.22	0.9991	1,488	3,363.84	2,369.67	0.427	2.25	0.9997
M7	2,039	3,291.46	2,987.86	0.0906	4.53	0.9990	2,312	3,257.41	3,126.58	0.083	4.69	0.9996
M15	2,005	3,000.73	2,858.48	0.0552	5.08	0.9999	2,270	3,230.46	3,072.73	0.050	5.23	0.9993
M30	2,083	2,852.41	2,809.60	0.0107	5.79	0.9990	2,355	3,074.52	3,019.14	0.009	5.924	0.9994
M60	2,191	2,980.65	2,976.04	0.0098	5.86	0.9996	2,471	3,129.46	3,118.24	0.0089	5.97	0.9994
M90	2,066	2,894.3	2,857.29	0.0078	6.06	0.9993	2,285	2,904.79	2,859.02	0.0073	6.15	0.9996
S7	1,625	3,459.65	2,637.44	0.2424	3.28	0.9992	1,783	3,146.64	2,592.74	0.233	3.38	0.9991
S15	1,905	2,899.67	2,797.22	0.0575	4.92	0.9991	2,106	3,202.16	2,859.32	0.053	5.04	0.9992
S30	2,064	2,745.75	2,758.83	0.0098	5.92	0.9993	2,324	2,886.78	2,879.97	0.008	6.04	0.9997
S60	2,023	2,811.02	2,802.89	0.0175	5.51	0.9992	2,257	2,894.26	2,881.14	0.015	5.61	0.9997
S90	2,084	2,940.73	2,910.22	0.0348	5.30	0.9996	2,365	3,171.65	3,101.03	0.031	5.45	0.9993

**FIGURE 2:**
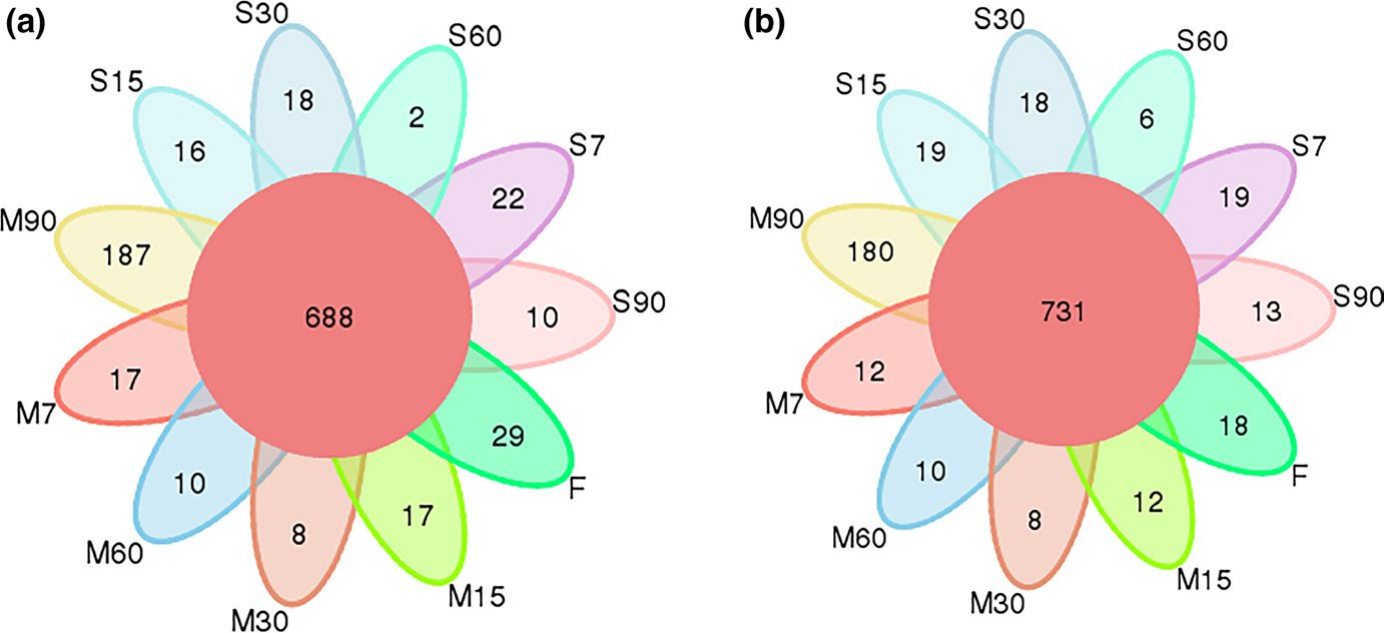
OTU cluster analysis of bacteria (a) and fungi (b) in red raspberry Enzymes. S refers to samples without starter cultures; M refers to samples with starter cultures. (7–90) indicate different fermentation stages (in days); F indicates fresh samples

Venn plots can be used to show the number of common and unique OTUs among samples. As shown in Figure 2, a total of 688 common bacterial OTUs and 731 fungal OTUs were obtained from all samples, while the numbers of unique OTUs were relatively low except for M90, for which the numbers of OTUs for bacteria and fungi were 187 and 180, respectively. In the entire fermentation process, the number of OTUs for common fungi was significantly higher than that for bacteria, but the number of OTUs for unique bacteria was slightly higher than that for fungi. Overall, the OTU numbers of fungi and bacteria were relatively adjacent, indicating that the two microorganisms were fully adapted to the fermentation environment of Enzymes.

### 3.3 Alpha diversity analysis

The diversity and richness of microbial communities can be reflected by alpha diversity analysis. The Shannon and Simpson indices are diversity estimators representing the approximated number of species and the evenness of their distributions in the samples (Dolores et al., 2017). Usually, a greater diversity of the community is accompanied by a higher Shannon index and lower Simpson index (Bai et al., 2015). As shown in Table 1, the Shannon index of bacteria and fungi in the M group gradually increased during the entire fermentation process, while the Simpson index gradually decreased, indicating that the diversity of microbial communities was highest in M90. However, the Shannon index of bacteria and fungi in the S group gradually increased within 30 days of fermentation and was relatively stable after this point. In addition, the higher Shannon index of fungi showed that the diversity of fungi was higher than that of bacteria.

The Chao and ACE indices are used to reflect the community richness. The higher the Chao and ACE indices are, the higher the community richness is (Bai et al., 2015). As shown in Table 1, the change in the Chao and ACE indices during the entire fermentation process was not irregular, while the Chao and ACE indices of bacteria in all samples, except M7 and S7, were slightly lower than those of fungi, indicating that the richness of fungi was higher.

Therefore, according to alpha diversity analysis, the diversity and richness of microbial communities in the M group increased as fermentation proceeded. These abundant microorganisms come not only from the red raspberry itself and initial starter cultures but also partly from the fermentation environment, such as fermentation barrels and other items that come into contact with the product surface. Moreover, compared to that of the S group, the diversity of bacteria and fungi in the M group was higher as fermentation progressed, indicating that the inoculated mixed strains (*L. plantarum* DQ‐13 and *K. ohmeri* WP) should have synergism with exogenous microorganisms from the environment. On the other hand, the diversity of bacteria and fungi in the S group slowly decreased during the later fermentation process, which may be related to the exogenous microorganisms not being suitable for the more acidic fermentation environment and the presence of chitin in the cell walls of fungi, which has a certain ability to resist environmental stress (Mei et al., 2016; Takenaka et al., 2014). The coverage, a sampling completeness indicator, had values >99.99% for bacteria and fungi (Table 1), indicating that sufficient bacterial and fungal diversity were discovered by the sampling regime used.

### 3.4 Microbial communities in red raspberry Enzymes

**FIGURE 3:**
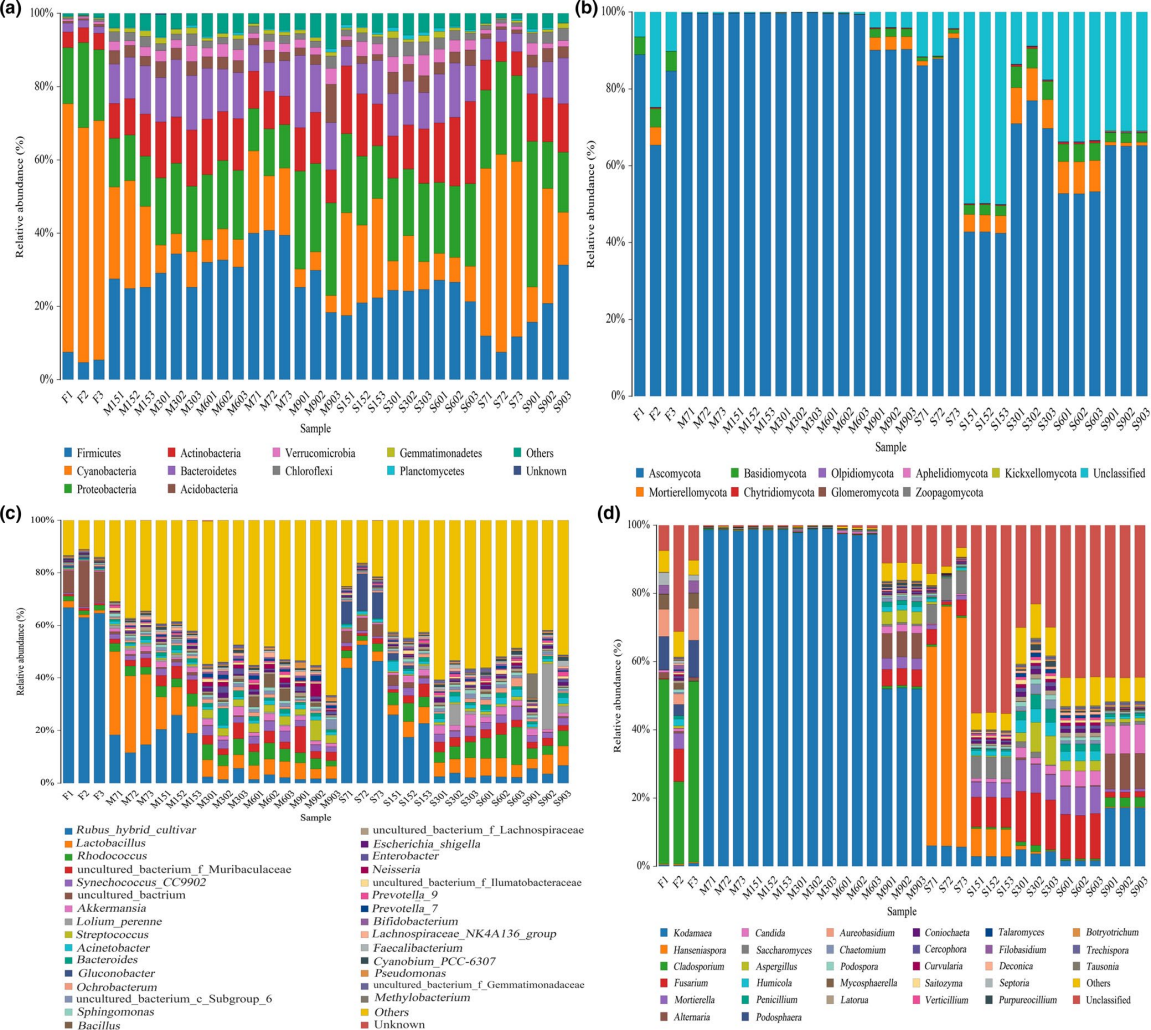
Relative abundance of bacteria (a) and fungi (b) at the phylum level and bacteria (c) and fungi (d) at the genus level in the samples. S refers to samples without starter cultures; M refers to samples with starter cultures. (7–90) indicate different fermentation stages (in days); F indicates fresh samples

The relative abundances of different bacterial microorganisms in all samples at the phylum level are shown in Figure 3a. Ten bacterial phyla, namely, Firmicutes, Actinobacteria, Verrucomicrobia, Gemmatimonadetes, Cyanobacteria, Bacteroidetes, Chloroflexi, Planctomycetes, Proteobacteria, and Acidobacteria, were identified according to the sequences obtained from all samples. There was a dramatic increase in the proportion of Firmicutes in the first week of fermentation, while the other members were present in minor percentages. Specifically, in the M samples, the proportion of Firmicutes increased to 40% by the first week and slightly decreased in the later fermentation process but was basically maintained at approximately 30%. In the S samples, the proportion of Firmicutes increased to 11.7% in the first week and then gradually increased to 26.1% after 60 days of fermentation. Microbial communities evolve from raw material mixing to the beginning of the ripening process, where the microbial composition becomes diverse and stable until the end of the process.

Figure 3b shows the relative abundance of fungi in all samples at the phylum level. All of the analyzed sequences were classified among 9 fungal phyla. Ascomycota represented the major fungal phylum in all samples, except for fresh samples and S samples that had been fermented for more than 15 days, which also had a high proportion of fungi from an unclassified phylum. The fungal community was dominated by the phylum Ascomycota, which is prevalent in fermented alcoholic beverages (XiaoRan et al., 2011). The remaining phyla, such as Basidiomycota and Mortierellomycota, also account for a certain proportion, which may play a role in the spontaneous fermentation process.

For a more detailed analysis of the bacterial and fungal dynamics associated with Enzymes fermentation, bacterial and fungal compositions at the genus level were determined. A total of 1,014 bacterial genera were detected during fermentation (the top 30 genera are shown in Figure 3c). In F samples and early fermented S samples, the gene *Rubus hybrid cultivar* was observed, which is a unique gene of red raspberry, and was amplified together when analyzing bacterial diversity, which may be related to the specificity of the universal 16S rRNA primers. Furthermore, *Lactobacillus* was the predominant genus during the early fermented stages in the M group, which is due to the inoculation of *L. plantarum* as a starter culture. *Lactobacillus* has been reported to produce several antimicrobial metabolites, including organic acids, which are related to the metabolism of amino acids during food fermentation processes (Quijada et al., 2017). Meanwhile, the ratio of *Lactobacillus* and *Rhodococcus* gradually increased during the subsequent fermentation process. However, the proportions of both genera fluctuated over the entire fermentation period. We speculate that this fluctuation was caused by changes in pH and alcohol content due to fermentation. Furthermore, the remaining major genera, *Akkermansia*, *Streptococcus*, *Prevotella*, *Acinetobacter*, etc., were present in our samples. Among them, *Acinetobacter* which can produce antibacterial substances has been reported (Wu et al., 2010). *Prevotella* has been found to have antitumor effects (Xin et al., 2019). However, their potential contribution to Enzymes is still unknown and requires further study. In addition, certain genera, such as *Enterobacter*, can cause recurrent bacteremia with strong antibiotic resistance. The production environment may become a secondary pollution source of *Enterobacter* (Wenzler et al., 2020), which is worth considerable attention and should not be regarded merely as a coincidence.

At the same time, 1,032 fungal genera were detected during fermentation (the top 30 genera are shown in Figure 3d). In the F group, the genus *Cladosporium* accounted for a relatively higher proportion (approximately 43.8%); this genus is an endophytic fungus of mangrove plants that can produce benzofuran derivatives and isocoumarin compounds through fermentation, with a certain antibacterial activity (Meng et al., 2017; Quaglia et al., 2020). In addition, *Cladosporium* can contribute a certain flavor and aromatic compounds to Enzymes in the early stage of fermentation and inhibit the production of other harmful microorganisms. In the M group, the genus *Kodamaea* dominated the fungal community because *K. ohmeri* WP‐1 was inoculated as a starter culture. *Kodamaea ohmeri* is often isolated from naturally fermented bacon, pickles, cheese, etc., representing the normal flora of fermented foods (Borelli et al., 2006; El‐Sharoud et al., 2009) and has the ability to degrade biogenic amines (Hui et al., 2014). After 90 days of fermentation, *Fusarium*, *Mycosphaerella,* and *Aspergillus* accounted for a certain proportion in M90, but *Kodamaea* was still the most abundant. The genus *Saccharomyces* also accounts for a certain amount in the S7 and S15 samples. *Saccharomyces* autolysis might release sugars, proteins, amino acids, organic acids, vitamins, and inorganic salt substances, providing carbon and nitrogen sources for fermentation and promoting the growth of other microorganisms during the late fermentation process. Some studies have reported that *Saccharomyces* commonly contributes to the flavor and aroma of fermented products (Vong & Liu, 2017). As fermentation progressed, the proportions of the initial primary genera decreased, and those of other genera, such as *Fusarium* and *Mortierella* increased. The main role of the genus *Fusarium* is speculated to involve the decomposition of cellulose and degradation of organic matter, promoting the circulation of biological substances in the fermentation environment. *Mortierella* can produce unsaturated fatty acids (Sakuradani et al., 2001), which are conducive to the formation of high‐quality products. After 90 days of fermentation, except for that of *Kodamaea*, the ratio of *Mycosphaerella* and *Candida* was increased in S90. *Candida* has been reported to produce a higher amount of flavoring substances than other yeasts by metabolizing branched‐chain amino acids through the Ehrlich pathway (O'toole, 1997). Other research results showed that *Candida* is predominant at the middle or late fermentation stage of products (Vanessa et al., 2014). Overall, Enzymes products inoculated with mixed starters have lower microbial diversity and abundance than those with spontaneous fermentation but are more controllable in terms of quality.

### 3.5 Comparison of the microbial communities in red raspberry Enzymes

**FIGURE 4:**
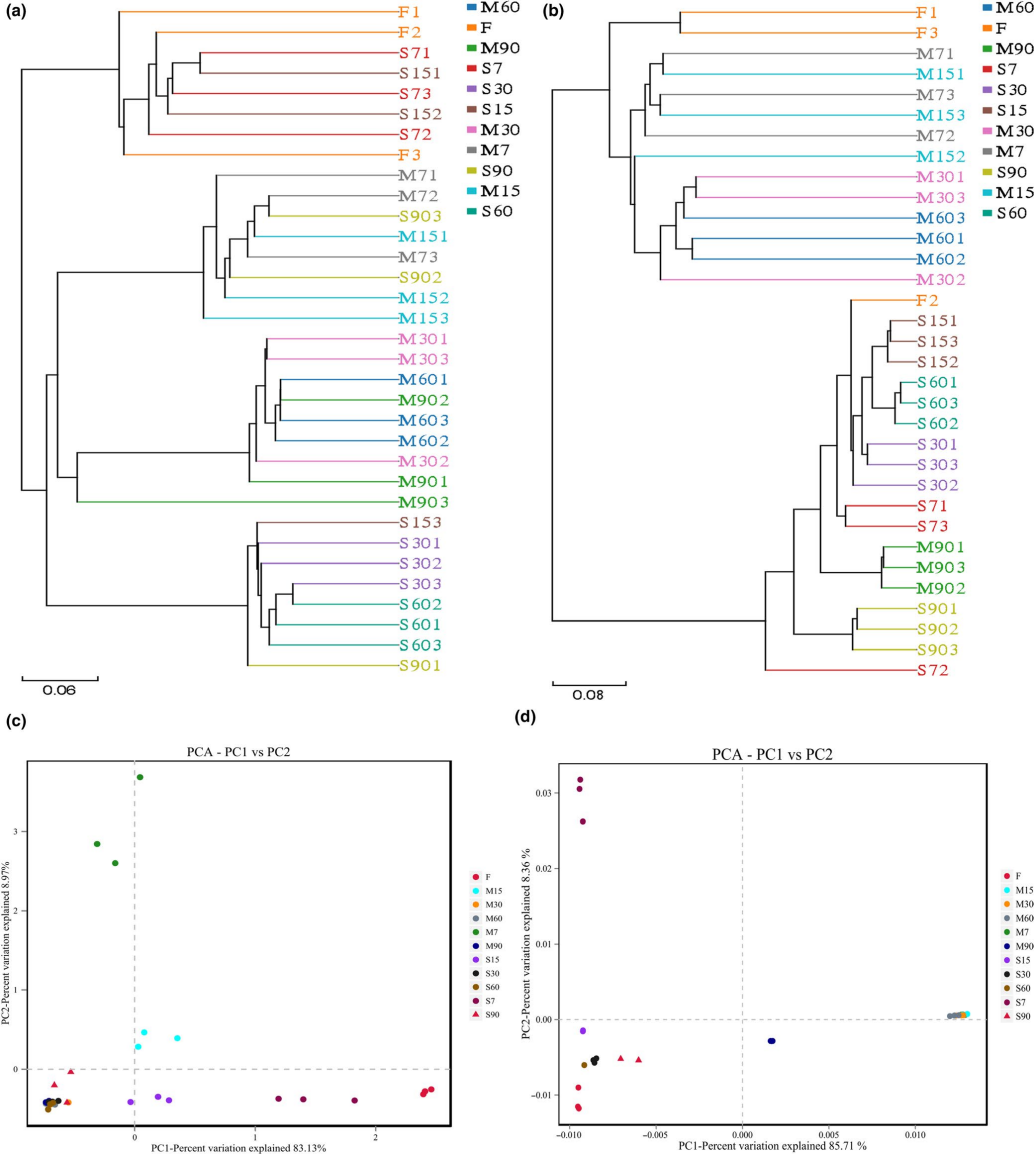
Unweighted pair‐group analysis (a) and principal component analysis (c) of the bacterial microbiota and unweighted pair‐group analysis (b) and principal component analysis (d) of the fungal microbiota in all samples. S refers to samples without starter cultures; M refers to samples with starter cultures. (7–90) indicate different fermentation stages (in days); F indicates fresh samples

Comparisons based on phylotype and phylogeny were performed to compare the bacterial and fungal compositions of samples collected at various stages of fermentation. Using a UPGMA tree considering phylotypic dissimilarity, combined with the 97% sequence identity of each sample, all samples at the bacterial level were split into four clades: Clade 1 comprised samples M30, M60, and M90; Clade 2 comprised samples M7, M15, S902, and S903; Clade 3 comprised samples S60, S30, S153, and S901; and Clade 4 comprised samples F, S7, S151, and S152 (Figure 4a). For comparison with the UPGMA clade results, principal component analysis (PCA) was used to determine the phylogenetic distances of the bacteria in each fermentation sample, and similar microbial clusters were obtained (Figure 4c). The first and second principal components (PCs) were shown to account for 83.13% and 8.97% of the cumulative percentage variance of species, respectively. In total, 92.1% of the species variances were explained by the two axes. In the M group, sample M7 was concentrated in the first quadrant, M15 was concentrated in the second quadrant, and the remaining samples were concentrated in the third and fourth quadrants, indicating that the difference in bacterial diversity between samples M7 and M15 was relatively significant, and the bacteria adapted to the environment of Enzymes during 15 days of fermentation. In the S group, S30, S60, and S90 were concentrated in the third quadrant. Since the S sample was naturally fermented, the microbial composition was more complicated, and the distribution was more concentrated in the later stage of the fermentation process.

Regarding the fungal community structures, all samples were also divided into four clusters (Figure 4b). M15, M30, and M60 were grouped together, and the corresponding PCA analysis was concentrated in the second quadrant (Figure 4d), which showed that the three groups have similar fungal microbial community structures. S15, S30, and S60 were dispersed in the third quadrant and were grouped together in the UPGMA analysis. The first and second principal components in the PCA analysis during Enzymes fermentation showed that the cumulative percentage difference of species accounted for 85.71% and 8.36%, respectively, and the two axes explained 94.07% of the species difference. The S7 sample was in the first quadrant alone, but it was grouped with S15, S30, and S60 in the UPGMA analysis, indicating that there is a certain difference between the two kinds of analysis, but the difference is not significant. In fact, almost all of the samples were separately spotted in the PCA, which suggested that specific microbial communities were present in each sample and that the microbiota was largely affected by fermentation time and starter cultures, which also confirms that there are more or less similar microbial populations in all samples.

### 3.6 The relationship between core species and antioxidant activity

**FIGURE 5:**
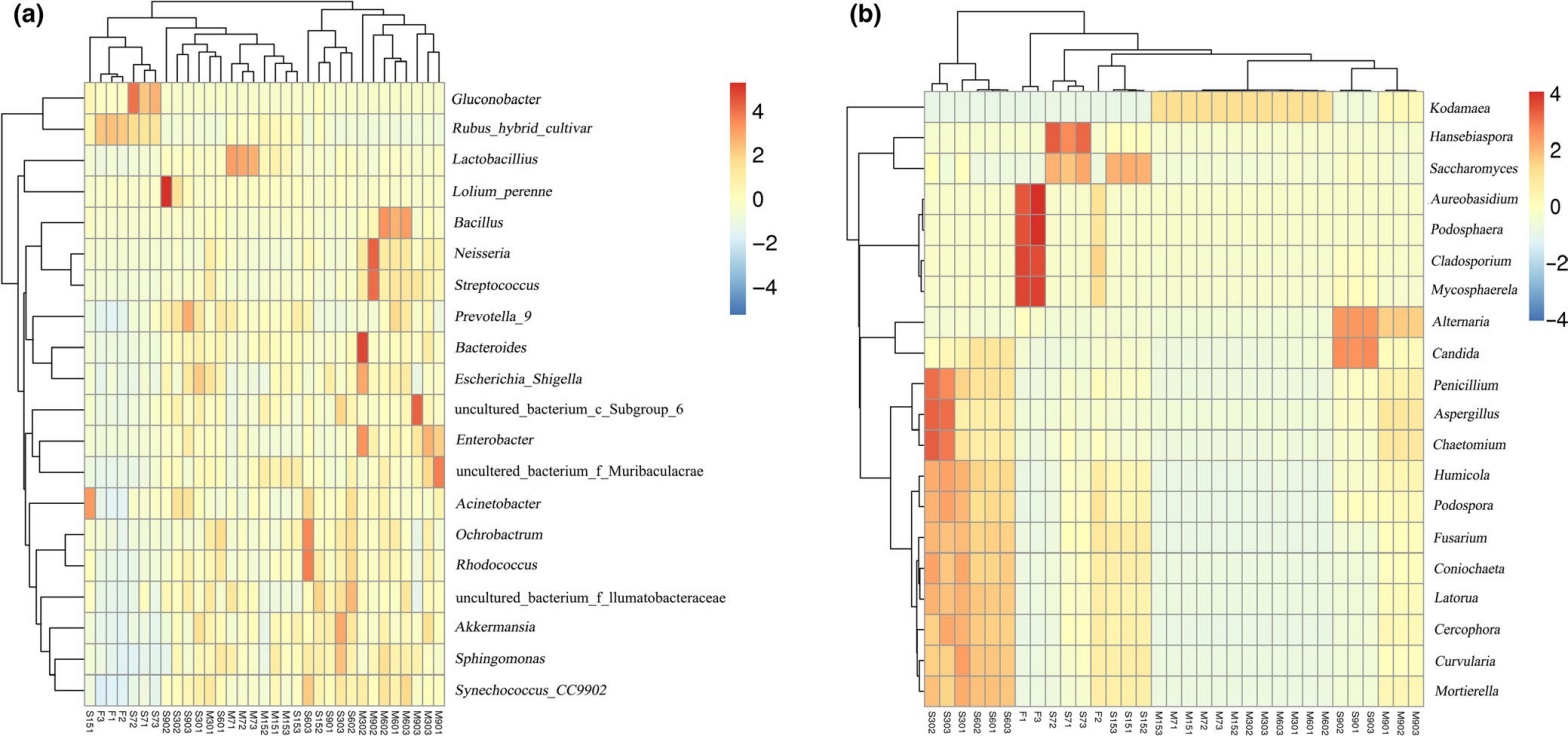
Bacterial community (a) and fungal community (b) heat map analysis at genus‐level phylotype. Each column in the heat map represents a different studied sample. S refers to samples without starter cultures; M refers to samples with starter cultures. (7–90) indicate different fermentation stages (in days); F indicates fresh samples

Heat maps expressing the relative abundance of microorganisms at the genus level were applied to visualize the community succession in the samples (Figure 5). In the heat maps, the redder and greener colors represented higher and lower relative abundances, respectively. The results showed that the dominant bacterial genera differed between the M and S groups during the fermentation process. For M samples, the dominant bacterial genera included *Bacteroides, Enterobacter, Lactobacillus, Bacillus, Streptococcus, and Neisseria*. The dominant fungal genera included *Alternaria, Candida, Setophoma, and Fusarium*. However, the main microbial species were complex in the S samples, with bacteria including *Ochrobactrum, Rhodococcus, Acinetobacter*, *Bacteroides, Prevotella_9, and Sphingomonas*. Fungi included *Hanseniaspora, Candida, Alternaria, Aspergillus, Penicillium, Fusarium*, and *Saccharomyces*. The use of starter cultures and lemons in Enzymes obviously decreased the levels of unknown microorganisms and inhibited the growth of many organisms associated with food spoilage (including pathogenic strains). For example, *Shigella* was reported to be the cause of chronic bacterial dysentery, mainly manifested as diarrhea (Elnaz et al., 2019). The reduction in undesired microbes and retention of dominant bacteria improved the microbiological quality and maintained the consistency of the products during the process.

**FIGURE 6:**
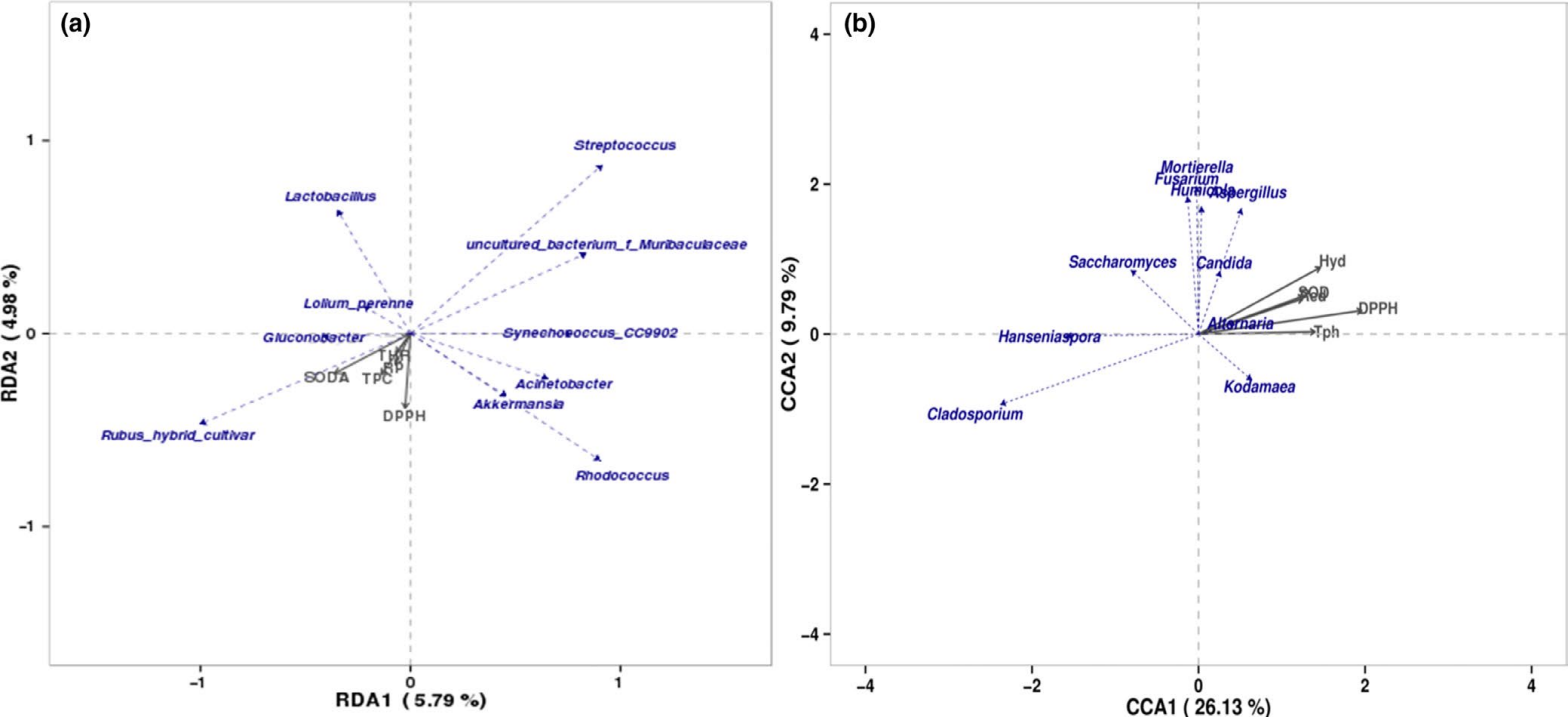
Correlation analysis of key bacteria (a) and fungi (b) and antioxidant activity

It is well known that the action of various microorganisms is probably responsible for the antioxidant activity in Enzymes (Young et al., 2014). Therefore, RDA and CCA were applied to further analyze the relationship between the specific species mentioned above and the antioxidant activity. RDA is based on a linear model, and CCA is based on a unimodal model. In general, according to the results of gradient lengths in the first axis through detrended correspondence analysis (DCA) of species OTUs, the best analysis model can be selected (Lindstrom et al., 2005). If the result is less than 3.0, RDA should be selected; if the result is greater than 4.0, CCA should be selected. Simultaneously, the closer the distance is, the closer the sample composition is; the relationship between rays is represented by the included angle. An acute angle indicates a positive correlation between the species and the environmental factors, while an obtuse angle indicates a negative correlation. As shown in Figure 6a, the total phenol content had a significant and positive correlation coefficient with *Gluconobacter* and a negative correlation coefficient with *Streptococcus* and *Lactobacillus*. Moreover, the SOD activity had a significant and negative correlation coefficient with *Synechococcus‐CC9902* and a positive correlation coefficient with *Gluconobacter*. The DPPH scavenging ability had a significant and positive correlation coefficient with *Rhodococcus* and *Akkermansia* and a negative correlation coefficient with *Streptococcus*. We speculate that the strain rapidly accumulated antioxidant components in the sample during the early stages of fermentation. In addition to the predominant species, other microorganisms are likely to contribute to the quality and antioxidant activity of Enzymes directly or indirectly, among the bacterial genera that possess a rather low relative abundance.

The results of fungal CCA (Figure 6b) showed that the total phenol content and DPPH scavenging ability were significantly positively correlated with *Kodamaea*, *Alternaria,* and *Candida* and that the SOD activity, hydroxyl radical scavenging ability, and reducing power all showed a significant positive correlation with *Fusarium* and *Mortierella*. The antioxidant activity index was negatively correlated with *Hanseniaspora* and *Cladosporium*. Combined with the CCA results and the previous antioxidant analysis, it can be seen that these fungi played an important role in improving the antioxidant activity of Enzymes during fermentation. Compared with other genera, *Saccharomyces* provides more flavor and volatile substances in the Enzymes. We speculate that the reason for the weak negative correlation between *Saccharomyces* and antioxidant substances is that some bacteria in the fermentation environment decompose macromolecules such as phenols into small molecules of sugar, which are widely used by *Saccharomyces*.

## 4 CONCLUSION

In conclusion, the antioxidant capacity of red raspberry Enzymes increases significantly as the fermentation time increases. In addition, the complex bacterial and fungal structures present in Enzymes were found to be very dynamic and varied with the time of the fermentation stage. Our results showed that the dominant microorganisms at the genus taxonomic level were identified, as well as the correlation between the microbial diversity and antioxidant activity of samples at different stages. There were significant differences in the bacterial and fungal community structure between the samples cultured with and without mixed starter, and the samples inoculated with mixed starter had higher antioxidant activity. The inoculation of mixed starters also significantly reduced the levels of unknown and undesired microorganisms. These results may help to provide a more controllable and useful theoretical framework for the development of multifunctional Enzymes products. Furthermore, the promotion mechanism of microbial metabolic pathways regarding the DPPH scavenging activity, SOD activity, and other antioxidant indicators needs further study.

## CONFLICT OF INTEREST

None of the authors of this study has any financial interest or conflict with industries or parties.

## ETHICAL APPROVAL

This study does not involve any human or animal testing.

## Data Availability

The data that support the findings of this study are available from the corresponding author upon reasonable request.
